# Pressure-enabled thyroid artery embolization: minimizing the risk of non-target embolization

**DOI:** 10.1186/s42155-026-00795-4

**Published:** 2026-11-14

**Authors:** Joe K. Khoury, Juan C. Camacho

**Affiliations:** 1 Department of Radiology and Imaging Sciences, Emory University School of Medicine, Atlanta, GA USA; 2 Department of Clinical Sciences, Florida State University College of Medicine, Sarasota, FL USA

## Abstract

Thyroid artery embolization (TAE) conventionally involves embolization of both superior thyroid arteries (STAs) and one inferior thyroid artery (ITA), preserving the contralateral ITA to maintain parathyroid perfusion. Selective STA catheterization carries risks of reflux-related non-target embolization and cerebral thromboembolism due to its origin from the carotid arterial system. Pressure-enabled TAE (PED-TAE) is an ITA-first technique that exploits intrathyroidal anastomoses between the STA and ITA to achieve ipsilateral lobar embolization without direct STA catheterization. The technique uses a valved microcatheter with a self-expanding braided nitinol mesh to reduce reflux and facilitate distal embolic delivery through pressure modulation. This technical letter provides a comprehensive, step-by-step guide to PED-TAE, covering preprocedural vascular assessment, arterial access and device selection, optimal microcatheter positioning, cone-beam CT evaluation, embolization technique, procedural endpoints, and peri- and postprocedural medication management. A practical workflow for patient selection and a procedural decision-making algorithm are presented to guide clinical decision-making, address anatomical and technical challenges, and facilitate reproducible application of the technique. Further prospective multicenter studies are warranted to establish the safety, efficacy, and long-term durability of PED-TAE relative to conventional TAE and surgical management.

**Supplementary Information:**

The online version contains supplementary material available at https://doi.org/10.1186/s42155-026-00795-4.

## Background

**Fig. 1:**
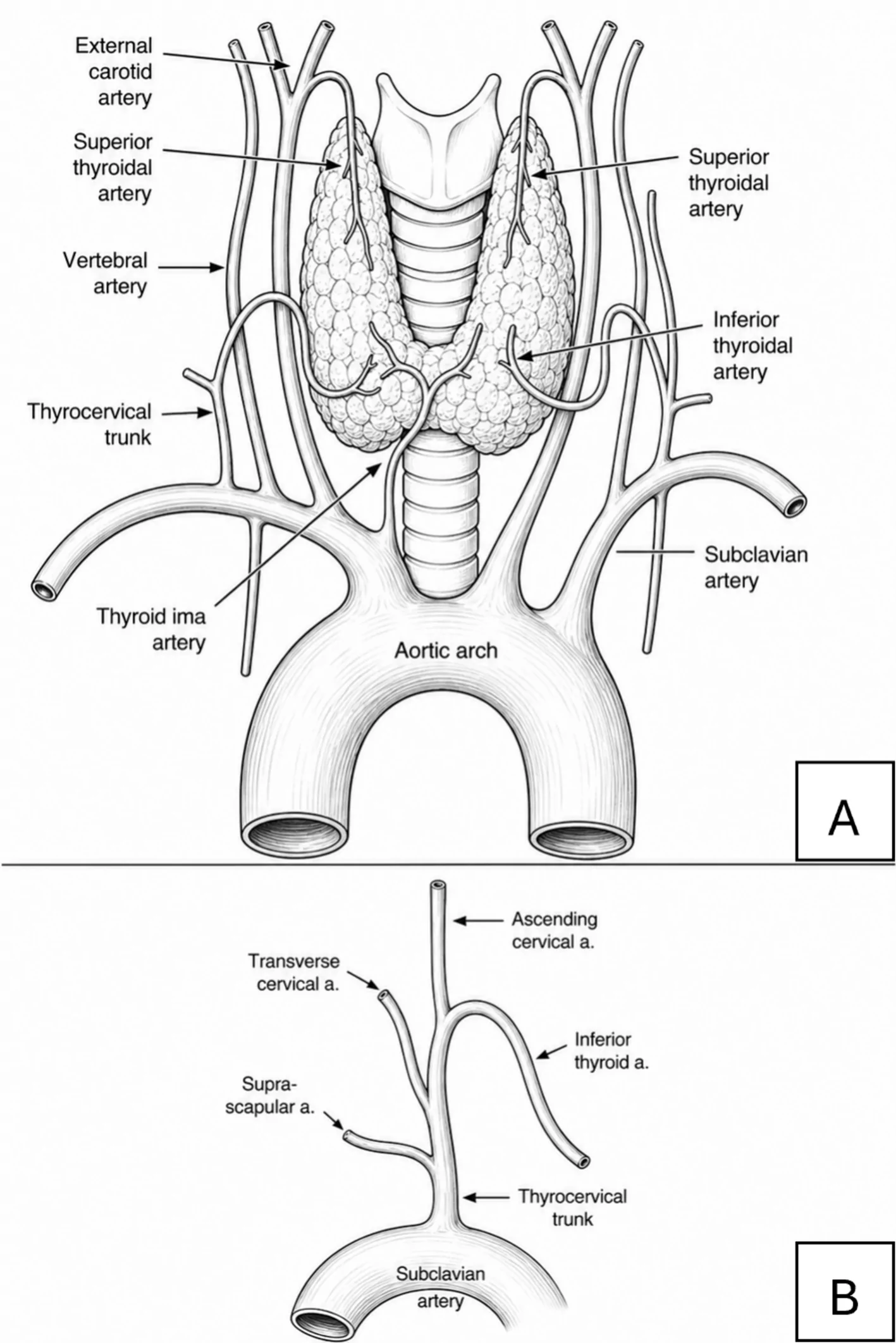
**A** Arterial vascular supply to the thyroid gland, depicting the inferior thyroid artery originating from the thyrocervical trunk off the subclavian artery, the superior thyroid artery originating from the external carotid artery, and a thyroid ima artery variant from the right innominate artery. **B** Arterial anatomy of the thyrocervical trunk, depicting the common branches proximal to the inferior thyroid artery

The first reported thyroid artery embolization (TAE) was performed in 1985 for the treatment of invasive medullary thyroid carcinoma [1]. Conventionally, TAE involves embolization of both superior thyroid arteries (STAs) and one inferior thyroid artery (ITA), preserving the contralateral ITA to maintain arterial perfusion to the parathyroid glands and theoretically reduce the risk of hypoparathyroidism. The STA most commonly arises from the proximal external carotid artery but may also originate from the common carotid artery or carotid bifurcation (Fig. 1A). Because of its origin within the carotid arterial system, selective STA catheterization carries inherent risks, including reflux-related non-target embolization and catheter-associated thromboembolism to the cerebral circulation. More recently, pressure-enabled thyroid artery embolization (PED-TAE) has been described as an alternative technique designed to reduce these risks [2]. PED-TAE takes advantage of the extensive intrathyroidal arterial anastomoses between the STA and ITA, allowing embolization of the entire ipsilateral thyroid lobe through the ITA alone. This is achieved using the TriNav microcatheter (TriSalus Life Sciences, Westminster, CO, USA), a pressure-enabled delivery device equipped with SmartValve™ technology. During ventricular systole, the valve collapses to permit antegrade blood flow at the microcatheter tip. During diastole, the valve expands radially to reduce reflux and facilitate pressure-enabled delivery of embolic material into the intrathyroidal arterial network. Unlike occlusive microcatheters, the TriNav permits antegrade blood flow when the valve is collapsed. To date, the TriNav is the only microcatheter described in the published literature for PED-TAE. Although other pressure-enabled or anti-reflux microcatheters may theoretically be suitable, their use for this technique has not yet been reported. By enabling embolization of the STA territory through the ITA, PED-TAE eliminates the need for direct STA catheterization and is intended to reduce the risks of reflux-related non-target embolization and cerebral thromboembolism while achieving complete ipsilateral lobar embolization. This manuscript builds on the initial description of PED-TAE by providing a comprehensive, step-by-step technical guide, from preprocedural planning through completion of embolization. The goal is to establish a practical, reproducible procedural framework for operators seeking to adopt this technique. Because PED-TAE remains an evolving approach, certain technical recommendations reflect the authors' procedural experience rather than findings from systematic evaluation. Further research is needed to standardize the technique and establish its safety, efficacy, and reproducibility.

## Technique

### Patient selection

Appropriate candidates for PED-TAE include patients with a large benign thyroid nodule greater than 30 mL, a multinodular goiter greater than 50 mL, a substernal or retrosternal goiter, a toxic multinodular goiter and those who are either not surgical candidates or require pre-operative embolization [3]. TAE can reduce goiter-related compressive symptoms and provide palliation in thyroid malignancies but does not replace surgery when indicated [3]. There are no procedure-specific absolute contraindications; relative contraindications are those generally associated with angiography, including pregnancy, iodinated contrast allergy, and chronic kidney disease [3]. Patient selection is comprehensively addressed in the recently published North American Society for Interventional Thyroidology (NASIT) statement, although vascular anatomy warrants additional consideration. Patients in whom the ITA is not visualized on CTA are not candidates for the ITA-based PED-TAE technique described in this manuscript. However, embolization via the STA remains an option for operators experienced with this approach. Patients with a history of thyroid thermal ablation may also be unsuitable for ITA-only PED-TAE. In our anecdotal experience, prior thermal ablation can disrupt intrathyroidal collateral pathways, preventing embolic delivery to the STA territory through the ITA. These patients may still undergo conventional thyroid artery embolization through separate catheterization of both the ITA and STA. Finally, patients with focal thyroid nodules identified on CTA may not achieve complete target coverage with ITA-only PED-TAE, as the nodules may receive independent arterial supply from both the ITA and STA or predominantly from the STA. In these cases, CBCT is essential to assess arterial supply and confirm adequate coverage of the target lesion before embolization.

### Planning and access

Pre-procedural CT angiography (CTA) of the head and neck is essential for delineating the vascular anatomy and planning PED-TAE. The ITA most commonly originates from the thyrocervical trunk (Fig. 1B), although anatomical variants may include an absent ITA, a thyroid ima artery arising from the brachiocephalic trunk or aortic arch, or aberrant/parasitized arterial supply from adjacent vessels, most commonly the internal mammary artery. These variants are encountered more frequently in large substernal goiters with mediastinal extension and should be identified pre-procedurally, as they may alter catheterization strategy and the feasibility or completeness of PED-TAE. CTA also defines thyroid gland anatomy and the distribution of disease, which can guide treatment laterality when mass effect or compressive symptoms are predominantly unilateral. In patients with a large solitary thyroid nodule, the dominant arterial supply may arise preferentially from either the ITA or STA, rather than through the extensive intrathyroidal arterial anastomoses typically seen in diffuse thyroid pathology, such as multinodular goiter or Graves' disease. Recognition of these anatomical and vascular features is critical for procedural planning and optimizing embolization strategy.

PED-TAE can be performed from femoral or radial artery access, although an ipsilateral radial approach is typically favored to minimize traversal of the supra-aortic vessels. If bilateral PED-TAE is performed, the preferred method is bilateral radial artery access to treat each side from an ipsilateral approach. Systemic heparinization and flush lines with heparinized saline should be utilized to limit catheter-associated thromboembolism.

**Fig. 2:**
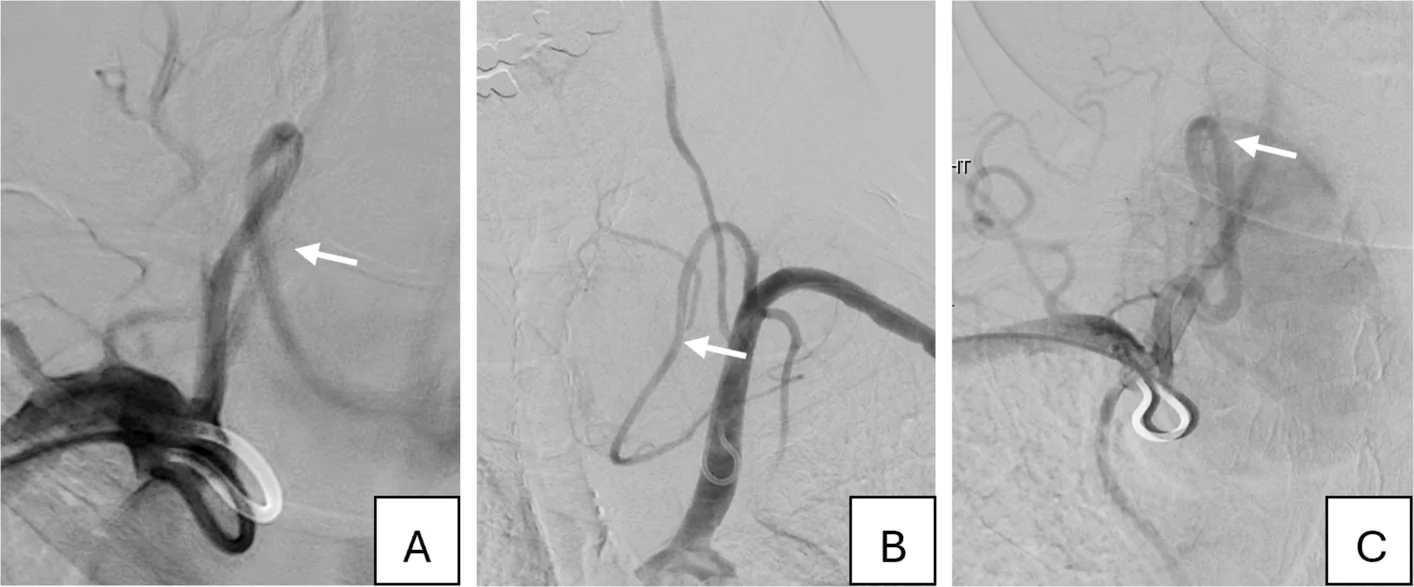
Digital subtraction angiography in the subclavian artery demonstrates 3 common anatomic morphologies of the inferior thyroid artery, resembling a cancer ribbon (**A**), candy cane (**B**), and diverging crossroads (**C**), respectively (white arrows)

**Fig. 3:**
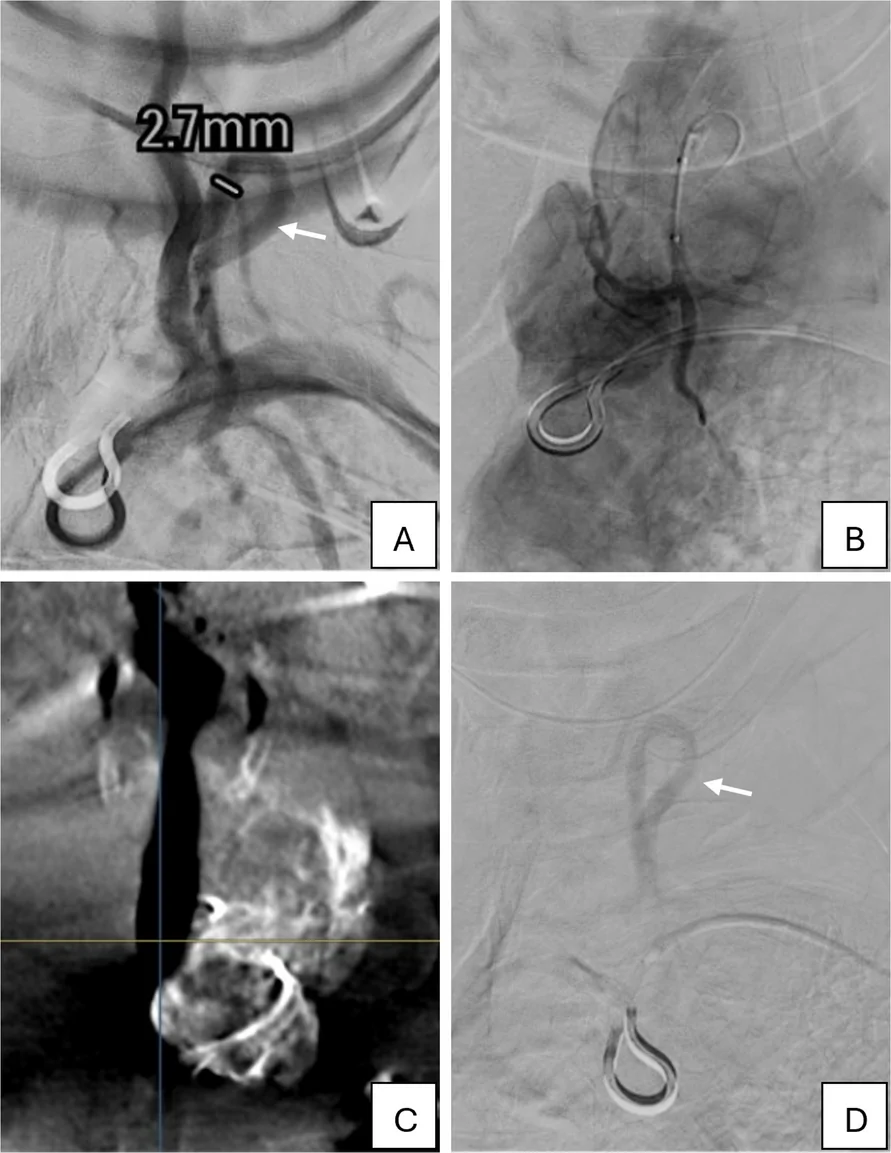
**A** Digital subtraction angiography (DSA) in the left subclavian artery demonstrates an enlarged inferior thyroid artery (ITA) (white arrow). The diameter is measured at its descending portion to determine appropriate pressure-enabled microcatheter size. This artery measured 2.7 mm and so the 2.4F TriNav FLX (TriSalus Life Sciences, Westminster, CO, USA) was used. **B** DSA with pressure-enabled microcatheter positioned in descending portion of the ITA, demonstrating enhancement of the entire left thyroid gland. **C** Cone-beam CT with pressure-enabled microcatheter in the ITA demonstrates enhancement of the entire left thyroid gland, including the superior thyroid artery territory. **D** DSA in the left subclavian artery with delayed imaging demonstrates patent inferior thyroid artery and thyrocervical trunk proximally without thyroid parenchymal enhancement, indicating an appropriate angiographic endpoint of embolization (white arrow)

After selecting the ipsilateral subclavian artery with a 5 F, 90–100 cm angled or reverse curve catheter, digital subtraction angiography (DSA) is performed with hand injection to identify the enlarged ITA arising from the thyrocervical trunk (Fig. 2A–C). The ITA diameter is then measured to determine the appropriate TriNav microcatheter (TriSalus Life Sciences, Westminster, CO, USA) to be used; 2.4 Fr if ≤ 3.5 mm or 2.9 Fr if > 3.5 mm vessel diameter (Fig. 3A). In smaller-caliber vessels, the ITA can often be selected directly with the base catheter by advancing a 0.014-inch microwire into the distal ITA branches. In larger vessels, a guide catheter with an inner diameter of at least 0.047 inches is required to accommodate the larger 2.9F microcatheter.

### Pressure-enabled delivery device positioning

After the microwire is advanced into the distal ITA branches to provide stable distal purchase, the TriNav microcatheter (TriSalus Life Sciences, Westminster, CO, USA) is advanced into the descending segment of the ITA and positioned distal to the first arterial turn, but just proximal to the intrathyroidal parenchymal branches (Fig. 3B). At this location, the catheter tip lies within the confines of the thyroid capsule, beyond the origin of proximal ITA branches, including the tracheoesophageal and ascending cervical arteries, thereby reducing the risk of non-target embolization. The microcatheter should also be positioned within a relatively straight segment of the vessel to ensure optimal function of its valve and maintain effective pressure-enabled antegrade embolic delivery.

### Digital subtraction angiography (DSA)/cone-beam CT (CBCT)

Once the microcatheter is in the desired position, power-injected DSA (e.g., 2 mL/s for a total of 8 mL at 1000–1200 psi) is performed to assess for reflux, identify non-target collateral flow, and confirm complete perfusion of the ipsilateral thyroid lobe (Fig. 3B). It is recommended to only perform power-injection DSA once the ITA is selected, to minimize risk of non-target air embolism. If the microcatheter is positioned too distally, incomplete opacification of the thyroid parenchyma may be observed, indicating that a portion of the gland is not being perfused. In this setting, the microcatheter can be withdrawn slightly to improve coverage, although catheter manipulation should be minimized because of the risk of vasospasm. Following satisfactory DSA, cone-beam CT (CBCT) is performed (e.g., 2 mL/s for a total of 22 mL with a 5-s x-ray delay) (Fig. 3C). The injection should be maintained throughout image acquisition so that the thyroid gland is imaged during the arterial parenchymal phase. A 5-s x-ray delay is generally appropriate because most CBCT systems require approximately 5–6 s to complete the 270° rotational acquisition, allowing continuous contrast opacification of the feeding arteries and thyroid parenchyma throughout the scan. CBCT should then be carefully reviewed to confirm complete perfusion of the ipsilateral thyroid lobe and to identify any potential non-target embolization pathways, including the vertebral, esophageal, muscular, and other cervical arterial branches.

### Embolization

Embolization is performed using 100–300 μm calibrated microspheres (Embosphere microspheres, Merit Medical Systems, South Jordan, UT, USA), as the average diameter of the intrathyroidal capillary and arteriolar anastomoses connecting the superior and inferior thyroid arteries ranges from approximately 120–250 μm, supporting the use of smaller embolic particles to traverse these collateral networks [4]. The pressure-enabled delivery microcatheter incorporates a self-expanding braided nitinol mesh that self-centers the catheter within the vessel and creates a partial flow restriction without completely occluding antegrade arterial flow [5–7]. During injection, this design increases local infusion pressure while minimizing reflux around the catheter tip, promoting preferential distal delivery and more uniform distribution of embolic particles throughout the thyroid vascular bed [5–7]. These altered flow dynamics facilitate deeper embolic penetration through the intrathyroidal anastomotic network, allowing embolization of both superior and inferior thyroid artery territories from a single ITA access. This ITA-first strategy is intended to reduce the risk of stroke by avoiding catheterization of the STA and leveraging the inherent anti-reflux properties of the microcatheter.

### Key angiographic findings and endpoints

**Fig. 4:**
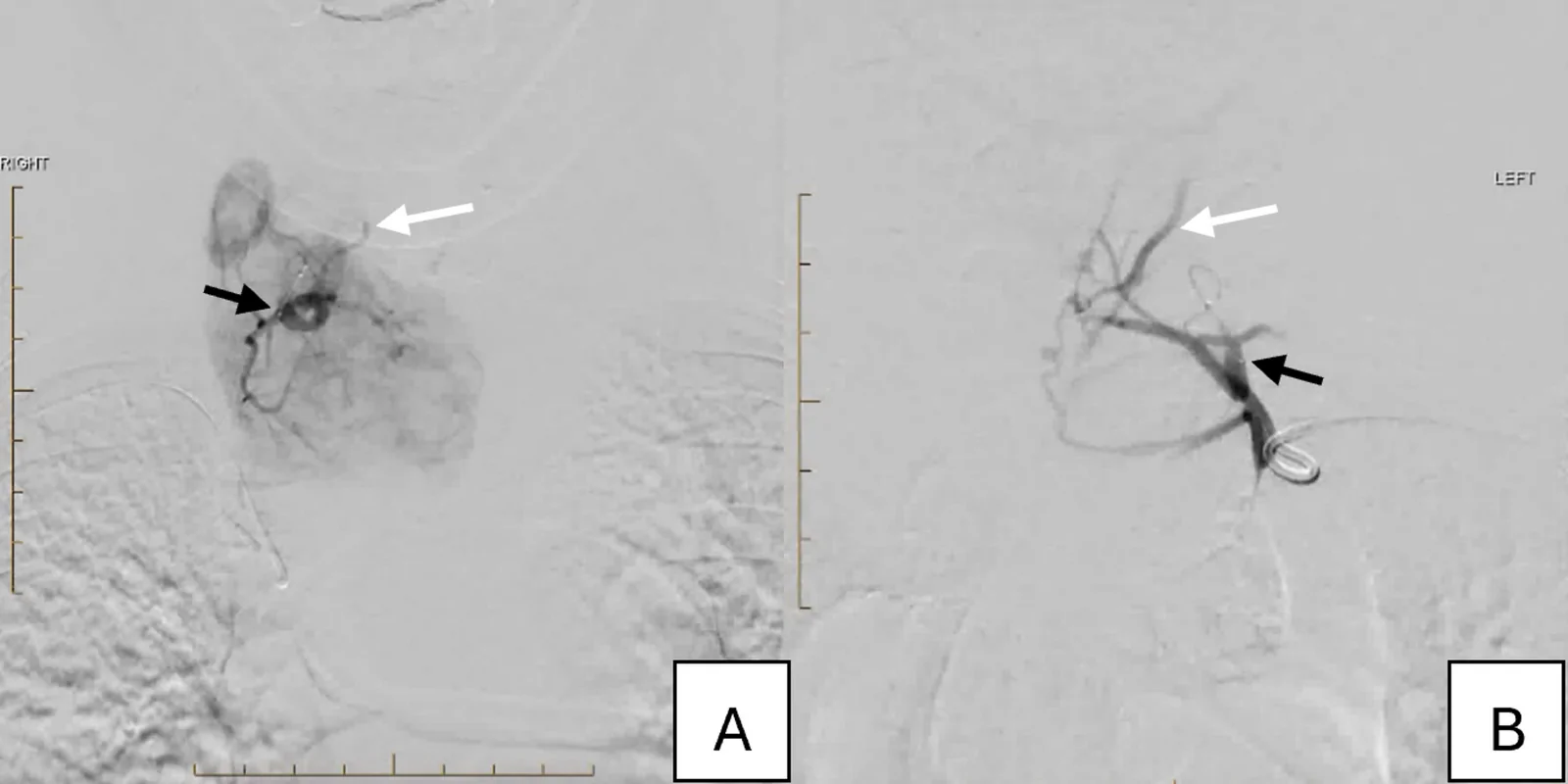
**A** Digital subtraction angiography (DSA) with a TriNav microcatheter (TriSalus Life Sciences, Westminster, CO, USA) in the right inferior thyroid artery (ITA) (black arrow) demonstrates pressure-mediated collateral recruitment and flow reversal in the superior thyroid artery (STA) (white arrow). **B** Post-embolization DSA with a TriNav microcatheter (TriSalus Life Sciences, Westminster, CO, USA) in the left ITA (black arrow) again shows pressure-mediated collateral recruitment and flow reversal in the superior thyroid artery (white arrow). *Post-embolization images were obtained prior to establishing protocol for pressure-enabled thyroid artery embolization. It is not recommended to do post-embolization angiography within the ITA, as there is a significant risk of non-target embolization and reflux of particles*

During embolization, fluoroscopic projections should be optimized to maximize visualization of the target vessel and facilitate early detection of reflux or non-target embolization. Slight contralateral rotation of the patient's head or adjustment to an oblique projection may improve visualization by reducing overlap from the clavicle or other osseous structures. Retrograde opacification of the STA may be observed through the intrathyroidal arterial anastomoses and should not be mistaken for reflux. This finding reflects the passage of contrast through the intrathyroidal microvascular network rather than retrograde migration of embolic particles, which are too large to traverse these distal capillary beds. If retrograde STA opacification occurs, temporarily pausing the injection allows restoration of antegrade flow before embolization is resumed. Throughout the embolization, the operator should continuously monitor for proximal reflux and the recruitment of non-target collateral vessels. Progressive parenchymal staining of the thyroid gland indicates successful embolic deposition. The angiographic endpoint is the development of a short column of stasis followed by slight microcatheter kick-back, reflecting increased resistance within the embolized vascular bed. After embolization, non-contrast CBCT should be performed to confirm diffuse retention of embolic material throughout the thyroid gland, demonstrated by persistent parenchymal staining. Post-embolization angiography should not be performed through the ITA or thyrocervical trunk, as forceful contrast injection may displace embolic particles and induce reflux (Fig. 4A–B). If required, a completion DSA can be obtained from the subclavian artery, demonstrating patency of the thyrocervical trunk and proximal ITA with minimal or absent thyroid parenchymal blush (Fig. 3D), if there is concern for suboptimal embolization when evaluating the post-embolization non-contrast CBCT. Complete contrast staining of the gland and confirmed endpoints can be used to skip the added risk of a post-procedure angiogram, as the likelihood of repeat embolization is minimal.

### Risk management

The goal of PED-TAE is controlled and safe devascularization of the thyroid while avoiding non-target embolization. Careful evaluation of the CTA prior to intervention is essential to choose a base catheter appropriate for the patient’s anatomy to avoid unnecessarily prolonged procedures. When selection of the thyrocervical trunk is difficult or not straightforward, consider aborting to avoid excessive catheter manipulation in close proximity to the vertebral artery. Avoid post-embolization angiography when not completely necessary to minimize the potential risk of particle reflux.

### Peri-procedural medication management

Beta blockade is recommended to minimize post-embolization transient hyperthyroidism. If the patient is not already on a beta blocker, 20 mg oral propranolol (Inderal, ANI Pharmaceuticals, Inc., Baudette, MN, USA) 1 week before and 4 weeks after embolization is suggested with a taper. Propranolol has the theoretical additional advantage of decreasing the conversion of active thyroid hormone in the peripheral bloodstream. Intravenous ketorolac (Toradol, Roche Laboratories Inc., Nutley, NJ, USA) 15 mg is administered before and after embolization for pain, and intravenous dexamethasone (Decadron, Merck & Co., Inc., Rahway, NJ, USA) 8 mg after embolization for inflammation. Be wary of intense peri-anal itching that can be experienced with intravenous dexamethasone. One gram intravenous acetaminophen (Ofirmev, Mallinckrodt Pharmaceuticals, Hazelwood, MO, USA) can be given after the procedure for continued pain management. A standard oral steroid taper is prescribed upon discharge.

## Conclusion

PED-TAE represents an evolution of conventional TAE. By combining pressure-enabled delivery with the thyroid gland's extensive intrathyroidal collateral circulation, embolization through a single ITA can often safely devascularize territories supplied by both the ITA and STA. Although thyroid artery embolization inherently carries a risk of stroke because of the proximity of the thyroid arteries to the carotid and vertebrobasilar circulations, PED-TAE is designed to mitigate this risk by avoiding catheterization of the carotid circulation and using a valved microcatheter that minimizes reflux with the advantage of improving distal parenchymal embolization. Prospective multicenter studies are needed to define the optimal technique, safety, efficacy, durability, and role of PED-TAE relative to conventional TAE and surgery.

## Supplementary Information

Supplementary Material 1. Supplemental Figure 1 A) Coronal CT demonstrates large multinodular goiter. B) 3D reconstruction depicts the extent of this goiter with significant substernal extension. C) Cone beam CT and D) digital subtraction angiography (DSA) with pressure-enabled microcatheter in the right inferior thyroid artery demonstrates enhancement of a large segment of the right thyroid gland, including the superior thyroid artery territory, but missing a large substernal nodule. E) DSA in the right innominate artery demonstrates a thyroid ima artery; an anatomic variant. F) Selective angiography within the thyroid ima artery shows enhancement of the previously missing thyroid nodule.

Supplementary Material 2: Supplemental Figure 2: Practical Procedural Workflow for Pressure-Enabled Delivery Thyroid Artery Embolization (PED-TAE).

Supplementary Material 3: Supplemental Figure 3: Decision Algorithm for Pressure-Enabled Delivery Thyroid Artery Embolization (PED-TAE).

Supplementary Material 4. Supplementary Video 1.

Supplementary Material 5. Supplementary Video 2.

## Data Availability

Not applicable.

## Abbreviations

TAE: Thyroid artery embolization
STA: Superior thyroid artery
ITA: Inferior thyroid artery
PED-TAE: Pressure-enabled delivery thyroid artery embolization
CTA: CT angiography
DSA: Digital subtraction angiography
CBCT: Cone-beam CT
